# Inhibition of TNF-α-Induced Collagen Degradation and Oxidative Damage by *Centipeda minima* and Brevilin A in Human Dermal Fibroblasts

**DOI:** 10.3390/cimb47050376

**Published:** 2025-05-20

**Authors:** Sullim Lee, Byoung Ha Kim, Yea Jung Choi, Dong-Wook Kim, Eunsu Cho, Moonseok Kang, Doeun Kim, Jaesung Pyo, Ki Sung Kang

**Affiliations:** 1 Department of Life Science, College of Bio-Nano Technology, Gachon University, Seongnam 13120, Republic of Korea; sullimlee@gachon.ac.kr; 2 D. Nature Co., Ltd., Seongnam 13174, Republic of Korea; mot37@d-nature.co.kr; 3 College of Korean Medicine, Gachon University, Seongnam 13120, Republic of Korea; domda22@gachon.ac.kr; 4 College of Pharmacy, Wonkwang University, Iksan 54538, Republic of Korea; pharmengin1@wku.ac.kr; 5 College of Pharmacy, Kyungsung University, Busan 48434, Republic of Korea; cho_es_99@naver.com; 6 Wonjin Plastic Surgery Clinic, Seoul 06735, Republic of Korea; flamekms@gmail.com; 7 Department of Pharmacology and Toxicology, Massachusetts College of Pharmacy and Health Sciences, Boston, MA 02115, USA; ehdms0801@gmail.com

**Keywords:** *Centipeda minima* (CMX), brevilin A, tumor necrosis factor-α (TNF-α), oxidative stress, extracellular matrix (ECM), skin aging, inflammation

## Abstract

Skin aging and inflammatory skin lesions are exacerbated by reactive oxygen species (ROS) generated in the mitochondria of human dermal fibroblasts (HDFs). These oxidative stressors degrade the extracellular matrix (ECM), promote inflammation, and accelerate skin aging. Antioxidants that suppress reactive oxygen species (ROS) production play a crucial role in mitigating these effects. This study investigated the protective effects of *Centipeda minima* (CMX) and its active constituent, brevilin A, against tumor necrosis factor-alpha (TNF-α)-induced oxidative stress and ECM degradation in normal human dermal fibroblasts (NHDFs). Both CMX and brevilin A significantly inhibited TNF-α-induced elevations in ROS, nitric oxide (NO), and prostaglandin E_2_ (PGE_2_) levels, thereby reducing oxidative stress and inflammatory responses. Additionally, they effectively suppressed matrix metalloproteinase-1 (MMP-1) expression and restored the procollagen I α1 (COLIA1) levels, indicating their potential to preserve ECM integrity. Mechanistically, brevilin A selectively inhibited ERK phosphorylation in the mitogen-activated protein kinase (MAPK) pathway, suggesting its role in regulating collagen degradation and inflammation. These findings highlight that CMX and brevilin A are promising natural agents for protection against skin aging and inflammation. However, further in vivo studies are necessary to validate their efficacy and explore their potential applications in dermatological formulations.

## 1. Introduction

The skin serves as the primary barrier that protects the internal organs from external environmental factors and plays a critical role in maintaining homeostasis. However, continuous exposure to intrinsic and extrinsic stressors leads to skin aging, a complex biological process characterized by structural and functional deterioration. Skin aging can be categorized into two types: intrinsic aging, which is driven by genetic and metabolic factors, and extrinsic aging, which results from environmental influences such as ultraviolet (UV) radiation, pollution, and chemical exposure. Intrinsic aging is associated with a gradual decline in cellular function, largely due to the accumulation of mitochondria-derived reactive oxygen species (ROS), which leads to oxidative stress and cellular damage [1,2]. In contrast, extrinsic aging is primarily driven by environmental factors, among which UV radiation is the most significant contributor [3,4].

Among the extrinsic factors, chronic exposure to UVA and UVB radiation accelerates skin aging, commonly referred to as photoaging. UV radiation generates excessive ROS in both epidermal and dermal cells, initiating oxidative stress, inflammation, and extracellular matrix (ECM) degradation [5,6]. These molecular events contribute to clinical manifestations such as wrinkles, hyperpigmentation, and loss of elasticity [1]. The overproduction of ROS results in oxidative damage to DNA, lipids, and proteins and activates matrix metalloproteinases (MMPs), particularly MMP-1, which degrade type I collagen and elastin in the ECM [7,8,9]. Consequently, prolonged UV exposure causes pronounced skin atrophy, deep wrinkles, and the overall deterioration of skin texture.

In addition to oxidative stress, UV radiation stimulates the production of pro-inflammatory cytokines, such as tumor necrosis factor-alpha (TNF-α), interleukins, and cyclooxygenase-2 (COX-2), as well as inflammatory mediators, such as inducible nitric oxide synthase (iNOS) [10,11]. TNF-α contributes to fibroblast dysfunction and upregulates collagen-degrading enzymes, thereby accelerating ECM breakdown and promoting visible signs of aging, such as sagging and wrinkle formation [12,13,14]. Moreover, chronic UV exposure sustains inflammation, which has been implicated in the pathogenesis of various skin disorders [5,6,15].

Given the deleterious effects of oxidative stress and chronic inflammation on the skin, increasing attention has been directed toward identifying bioactive compounds that can attenuate ROS generation and prevent ECM degradation. In particular, natural compounds with antioxidant and anti-inflammatory properties have been explored for their potential to counteract TNF-α-induced skin damage [16,17]. Many plant-derived phytochemicals have demonstrated their ability to inhibit ROS accumulation, suppress MMP-1 expression, and restore collagen synthesis, highlighting their potential in preventing or delaying skin aging [18,19].

*Centipeda minima* (L.) Braun and Asch., which are medicinal plants belonging to the Asteraceae family, are widely distributed across East Asia (China, Korea, and Japan), Oceania (Australia), and Southern Asia (India). They have been traditionally used in Chinese medicine to treat various ailments, including headaches, coughs, colds, nasal allergies, asthma, diarrhea, and malaria. Recent pharmacological studies have reported that *C. minima* extracts exhibit antioxidant, anti-inflammatory, antibacterial, neuroprotective, and anticancer properties [20,21]. These pharmacological properties have been attributed to their rich phytochemical compositions, and several active compounds have been identified and quantified.

In our previous study, we investigated the effects of *C. minima* extracts on human hair follicle dermal papilla cells (HFDPCs) and elucidated the hair growth-promoting mechanism. Using high-performance liquid chromatography coupled with quadrupole time-of-flight mass spectrometry (HPLC-Q-TOF-MS), we identified four major bioactive sesquiterpene lactones: arnicolide D, arnicolide C, micro-helenin C, and brevilin A [20,21]. Among these, brevilin A exhibited the strongest biological activity, particularly in antioxidant assays. However, the protective effects of *C. minima* and its active constituents against TNF-α-induced oxidative stress and inflammation in skin cells remain unclear.

Given that oxidative stress and inflammatory responses are major contributors to skin aging and related disorders, natural agents capable of modulating these processes are of significant interest for dermatological therapeutics and cosmetics. In particular, the inhibition of ROS production and MMP-1 activity is a key strategy in preventing ECM degradation. Considering the potent antioxidant and anti-inflammatory properties of *C. minima* and brevilin A, these compounds may have protective effects against TNF-α-induced skin damage.

To address this research gap, we aimed to investigate the protective effects of *C. minima* extract (CMX) and its active compound brevilin A against TNF-α-induced oxidative stress and ECM degradation in normal human dermal fibroblasts (NHDFs). Specifically, we examined their ability to suppress ROS production, inhibit pro-inflammatory mediators such as nitric oxide (NO) and prostaglandin E2 (PGE2), regulate MMP-1 expression, and restore procollagen I α1 (COLIA1) levels. In addition, we explored their effects on mitogen-activated protein kinase (MAPK) signaling, with a focus on ERK phosphorylation, to elucidate the potential molecular mechanisms. The findings of this study provide valuable insights into the potential use of *C. minima* and brevilin A as natural agents to prevent skin aging and inflammatory skin conditions.

## 2. Materials and Methods

### 2.1. Plant Materials and Preparation of CMX

**Figure 1:**
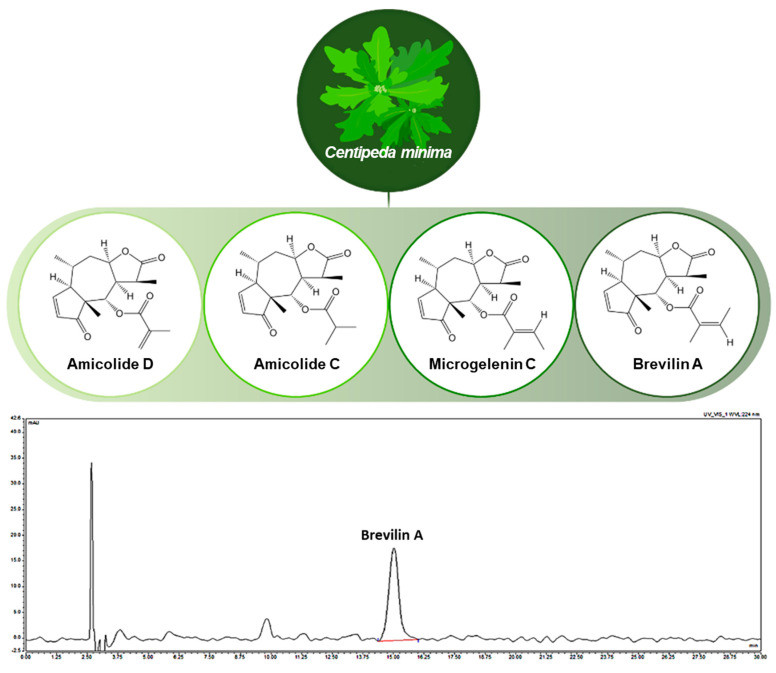
Chemical structures of active compounds in *Centipeda minima* and high-performance liquid chromatography chromatogram of CMX.

*Centipeda minima* (CMX) was obtained as a natural herb from Goesan, South Korea, in December 2019. The dried aerial parts were pulverized and extracted with 70% ethanol at 60 °C for 3 h using a reflux extractor. The extract was filtered, evaporated under reduced pressure, and lyophilized to obtain a powder. The primary bioactive constituents of CMX include arnicolide D, arnicolide C, microgelenin C, and brevilin A, with their chemical structures depicted in Figure 1. Extraction and analysis were performed according to previously described methods [20,21].

### 2.2. Cell Culture and Sample Preparation

Normal human dermal fibroblasts (NHDFs) derived from juvenile foreskin were purchased from PromoCell GmbH (Heidelberg, Germany). Cells were cultured in Dulbecco’s Modified Eagle’s Medium (DMEM; Gibco, Waltham, MA, USA) supplemented with 10% fetal bovine serum (FBS; Gibco, Waltham, MA, USA) and 1% penicillin–streptomycin (100 U/mL penicillin and 100 μg/mL streptomycin) at 37 °C in a humidified 5% CO_2_ incubator. Cells were passaged at 70–80% confluence and used between passages three and six. CMX and brevilin A were dissolved in DMSO and diluted in media to final concentrations ranging from 1 to 100 μg/mL (CMX) and 0.1 to 10 μM (brevilin A), with the final DMSO concentration kept below 0.1%.

### 2.3. Cell Viability

NHDFs were seeded at 1 × 10^4^ cells/well in 96-well plates and incubated overnight. Cells were treated with CMX (1, 10, 30, 100 μg/mL) or brevilin A (0.1, 0.5, 1, 5, 10 μM) for 24 h. EZ-Cytox solution (10 μL) was added to each well and incubated for 2 h. The absorbance was measured at 450 nm using a SpectraMax microplate reader. Cell viability was expressed relative to that of untreated controls. The experimental details are similar to those of Lee et al. [22].

### 2.4. Measurement of Intracellular ROS

Intracellular ROS production was measured using DCFDA (Sigma-Aldrich, St. Louis, MO, USA). NHDFs were seeded at 1 × 10^4^ cells/well in black 96-well plates and were incubated overnight. The cells were pretreated with CMX or brevilin A for 1 h and then stimulated with TNF-α (10 ng/mL) for 1 h. After stimulation, the cells were washed and incubated with 25 μM DCFDA in serum-free medium for 30 min at 37 °C. Fluorescence was measured (excitation: 485 nm, emission: 535 nm) using a multimode plate reader. ROS levels were normalized to those of TNF-α-treated controls.

### 2.5. Measurement of Nitric Oxide (NO) Production

NO production was assessed using the Griess reagent assay. NHDFs were seeded at 1 × 10^4^ cells/well in 96-well plates, pretreated with CMX or brevilin A for 1 h, and stimulated with TNF-α (10 ng/mL) for 24 h. The culture supernatants (50 μL) were mixed with an equal volume of Griess reagent (1% sulfanilamide and 0.1% NED in 2.5% phosphoric acid) and incubated for 10 min at room temperature. Absorbance was measured at 540 nm. Results were calculated using a sodium nitrite standard curve.

### 2.6. Measurement of Protein Secretion

NHDFs were seeded at 2 × 10^4^ cells/well in 48-well plates and were incubated overnight. The cells were pretreated with CMX (10 or 30 μg/mL) or brevilin A (0.5 or 1 μM) for 1 h and then stimulated with TNF-α (10 ng/mL) for 24 h. The supernatants were collected and the levels of MMP-1, COLIA1, and PGE2 were quantified using ELISA kits (R&D Systems, Minneapolis, MN, USA), following the manufacturer’s protocols. The results are expressed as pg/mL or ng/mL, based on standard curves.

### 2.7. Western Blotting

Western blotting was performed to assess the expression of mitogen-activated protein kinases (MAPKs), including extracellular signal-regulated kinase (ERK), c-Jun N-terminal kinase (JNK), and p38. NHDFs were seeded at a density of 3 × 10^5^ cells per well in a 6-well plate and cultured overnight. The cells were pretreated with CMX and brevilin A for 1 h, followed by TNF-α stimulation for the indicated times. After treatment, cells were lysed using RIPA buffer supplemented with protease and phosphatase inhibitors. The protein concentration was determined using the bicinchoninic acid (BCA) assay. Equal amounts of protein were separated by SDS-PAGE and transferred onto polyvinylidene difluoride (PVDF) membranes using the wet-transfer method. Membranes were blocked with 5% skim milk and incubated overnight with primary antibodies against ERK, JNK, and p38, followed by incubation with horseradish peroxidase (HRP)-conjugated secondary antibodies. Protein bands were visualized using enhanced chemiluminescence (ECL) and analyzed using the ImageJ software (1.54f). The detailed Western blot procedure followed a previously established protocol. 

### 2.8. Statistical Analysis

All experiments were performed in triplicate, and data are expressed as the mean ± standard error of the mean (SEM). Statistical significance was determined using one-way analysis of variance (ANOVA), followed by Tukey’s post hoc test for multiple comparisons. Statistical significance was set at *p* < 0.05. Statistical analyses were conducted using the GraphPad Prism software (v10.4.1, GraphPad Software Inc., San Diego, CA, USA).

## 3. Results

### 3.1. Content of Brevilin A in CMX

#### Effects of CMX and Brevilin A on Intracellular ROS Accumulation and MMP-1 and COLIA1 Secretion in TNF-α-Stimulated NHDFs

**Figure 2:**
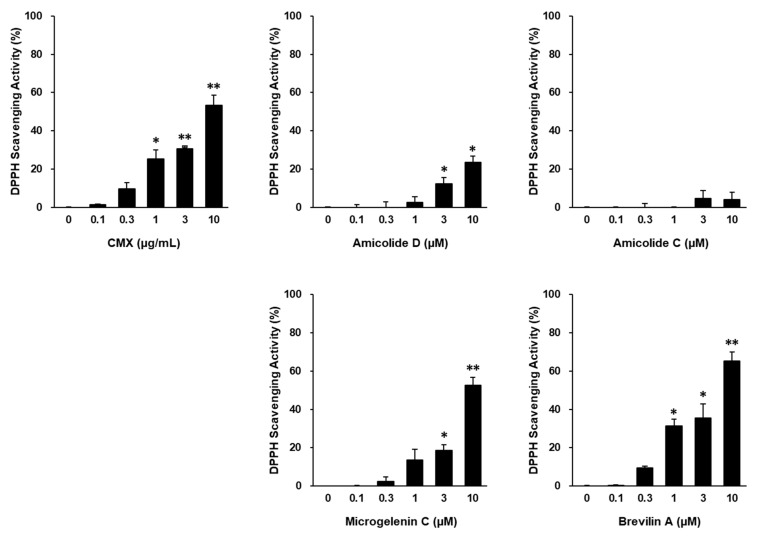
Antioxidant effects of CMX, amicolide D, amicolide C, microgelenin C, and brevilin A. DPPH radical scavenging activity for the indicated treatments was calculated based on the ratio to the control group. Results are presented as the mean ± standard error of the mean (SEM). * p < 0.05 and ** p < 0.01 vs. control group.

The extract of *C. minima* (CMX) exhibited significant DPPH radical scavenging activity, indicating its strong antioxidant potential (*p* < 0.05, 1 μg/mL; *p* < 0.01, 3, and 10 μg/mL). Among the four major bioactive compounds identified in CMX, brevilin A showed the most potent antioxidant effects (*p* < 0.05, 1 and 3 μM; *p* < 0.01, 10 μM) (Figure 2).

**Figure 3:**
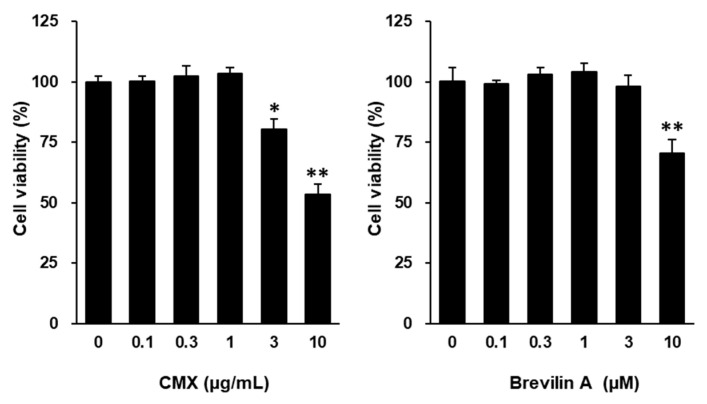
Effects of CMX and brevilin A on cell viability of NHDFs. NHDFs were seeded in 96-well plates at a density of 1 × 10^4^ cells/well and cultured for 24 h before being exposed to serum-free conditions. After starvation for at least 16 h, cells were exposed to the indicated treatments. Cell viability was measured using EZ-Cytox. Cell viability for the indicated treatments was calculated based on the ratio to the vehicle group (DMSO), which was set to 100%. Results are presented as the mean ± standard error of the mean (SEM). * *p* < 0.05 and ** *p* < 0.01 vs. vehicle group.

Given its superior activity, brevilin A was selected as the primary active ingredient responsible for the protective effects of CMX against oxidative stress-induced skin damage (Figure 3). Based on the preliminary toxicity assessments, CMX and brevilin A were used at concentrations of 1 μg/mL and 3 μM or lower, respectively, for subsequent experiments.

**Figure 4:**
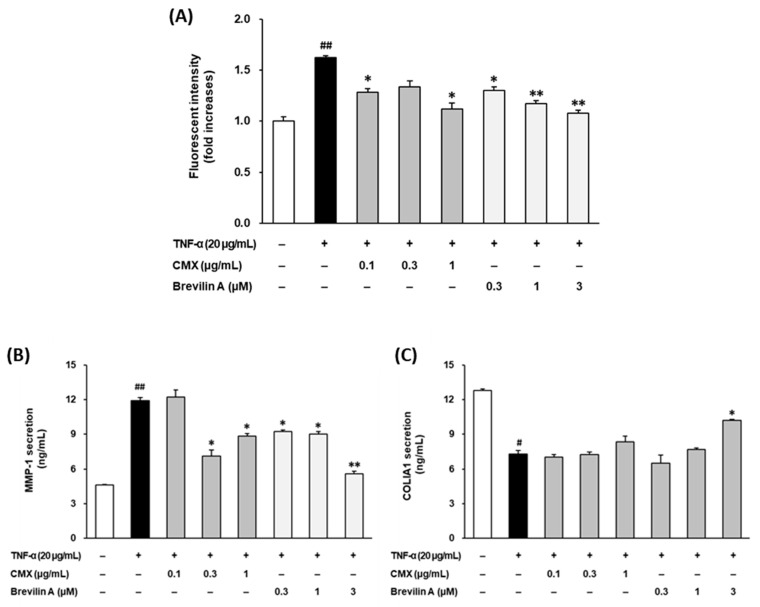
Effects of CMX and brevilin A on ROS accumulation and MMP-1 and COLIA1 secretion. (**A**) NHDF cells were dispensed into each well at a concentration of 1 × 10^4^. To synchronize the cell cycle, cells were incubated in FBS-free media for 24 h. Subsequently, brevilin A and CMX were added at various concentrations, followed by TNF-α and DCFDA. The cells were incubated for 15 min and the fluorescence values were measured. (**B**,**C**) HDF cells were dispensed into each well of a 48-well cell culture plate at a concentration of 2 × 10^4^. The cell cycle was synchronized by incubating the cells in FBS-free medium. Brevilin A and CMX were added to each well at various concentrations, followed by treatment with TNF-α. After 24 h of incubation, the supernatants were collected. The proteins present in the supernatant were measured by ELISA. (**A**–**C**) The experimental results are presented as S.E.M. Statistical significance is indicated as follows: *p* < 0.05 (#) and *p* < 0.01 (##) indicate significance compared to the control group, while *p* < 0.05 (*) and *p* < 0.01 (**) indicate significance compared to the TNF-α-induced group.

To investigate the potential of CMX and brevilin A to mitigate oxidative stress, the intracellular ROS levels were measured in TNF-α-stimulated NHDFs using DCFDA fluorogenic dye. As shown in Figure 4A, TNF-α stimulation significantly increased ROS production by 1.62 ± 0.02-fold compared to the control group (*p* < 0.05). Treatment with CMX effectively reduced ROS accumulation in a dose-dependent manner, with concentrations of 0.1, 0.3, and 1 μg/mL showing significant inhibition (*p* < 0.05, 0.1, and 1 μM).

Similarly, 0.1, 0.3, and 1 μM brevilin A suppressed ROS generation, with a significant reduction observed at 0.3 μM and higher concentrations (*p* < 0.05 for 0.3 μM; *p* < 0.01 for 1 and 3 μM). Notably, CMX (1 μg/mL) and brevilin A (1 μM) restored the ROS levels to levels comparable to those in the vehicle-treated control group. These findings indicated that CMX and brevilin A effectively mitigated oxidative stress by reducing ROS overproduction in TNF-α-stimulated NHDFs.

To evaluate the protective effects of CMX and brevilin A on ECM degradation, the secretion of MMP-1, a key enzyme responsible for collagen breakdown, was assessed by ELISA (Figure 4B). TNF-α treatment significantly increased MMP-1 secretion to 11.9 ± 0.25 ng/mL, compared to 4.60 ± 0.06 ng/mL in the untreated control group (*p* < 0.01). Treatment with CMX at 0.3 and 1 μg/mL reduced MMP-1 secretion to 7.10 ± 0.57 ng/mL and 8.85 ± 0.22 ng/mL, respectively (*p* < 0.05). Similarly, brevilin A at concentrations of 0.1, 0.3, and 1 μM effectively suppressed MMP-1 secretion to 9.25 ± 0.09 ng/mL, 9.02 ± 0.20 ng/mL, and 5.60 ± 0.19 ng/mL, respectively (*p* < 0.05 for 0.1 and 0.3 μM; *p* < 0.01 for 1 μM). These results demonstrate that both CMX and brevilin A protected against TNF-α-induced ECM degradation by inhibiting MMP-1 secretion.

To further examine the impacts of CMX and brevilin A on ECM integrity, the secretion of procollagen I α1 (COLIA1), a precursor of collagen type I, was measured (Figure 4C). TNF-α stimulation significantly reduced COLIA1 secretion from 12.8 ± 0.13 pg/mL to 7.30 ± 0.32 pg/mL (*p* < 0.05), confirming its role in collagen degradation. Treatment with CMX at 1 μg/mL increased COLIA1 secretion to 8.35 ± 0.47 pg/mL, although the effect was not statistically significant. In contrast, brevilin A at 3 μM significantly restored COLIA1 secretion to 10.2 ± 0.06 pg/mL, with statistical significance observed at 3 μM (*p* < 0.05). These findings suggest that brevilin A plays a crucial role in preserving collagen synthesis and ECM integrity in TNF-α-stimulated NHDFs.

### 3.2. Effects of CMX and Brevilin A on NO and PGE_2_ Overproduction in TNF-α-Stimulated NHDFs

To evaluate the anti-inflammatory effects of *C. minima* (CMX) and brevilin A, the production of nitric oxide (NO) and prostaglandin E_2_ (PGE_2_) was measured in TNF-α-stimulated NHDFs.

**Figure 5:**
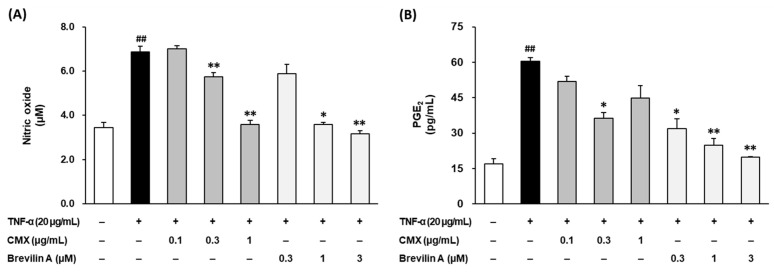
Effects of CMX and brevilin A on pro-inflammatory NO (**A**) and PGE_2_ (**B**). HDF cells were dispensed into a 96-well plate at a concentration of 1 × 10^4^. To synchronize the cell cycle, the medium was replaced with FBS-free medium and the cells were incubated for 24 h. The cells were then pretreated with various concentrations of brevilin A and CMX, followed by the addition of TNF-α. After 24 h of incubation, the supernatants were collected. The collected supernatant was used to measure the NO and PGE_2_ levels. The experimental results are presented as S.E.M., and statistical significance is indicated as follows: *p* < 0.01 (##) compared to the control group and *p* < 0.05 (*) and *p* < 0.01 (**) compared to the TNF-α-induced group.

The NO levels in the culture supernatants were quantified using the Griess reagent assay. As shown in Figure 5A, TNF-α stimulation significantly increased NO production to 6.87 ± 0.26 μM compared to 3.45 ± 0.24 μM in the untreated control group (*p* < 0.01). Treatment with CMX significantly reduced the NO levels in a dose-dependent manner, with 0.3 μg/mL decreasing NO to 5.75 ± 0.19 μM (*p* < 0.01) and 1 μg/mL reducing NO to 3.59 ± 0.18 μM (*p* < 0.01). Similarly, brevilin A treatment at 0.3 and 1 μM effectively suppressed NO production to 3.59 ± 0.09 μM (*p* < 0.05) and 3.17 ± 0.13 μM (*p* < 0.01), respectively. These results indicate that both CMX and brevilin A effectively inhibited TNF-α-induced NO overproduction in NHDFs, suggesting their potential role in reducing oxidative stress and inflammatory responses.

To further examine the anti-inflammatory effects of CMX and brevilin A, the PGE_2_ levels in the culture supernatant were measured using ELISA. As depicted in Figure 5B, TNF-α stimulation significantly increased PGE_2_ production from 16.9 ± 2.21 pg/mL in the control group to 60.5 ± 2.15 pg/mL (*p* < 0.01). Treatment with CMX at 1 μg/mL significantly reduced the PGE_2_ levels to 36.3 ± 2.47 pg/mL (*p* < 0.05). Similarly, brevilin A suppressed the PGE_2_ levels in a dose-dependent manner, with reductions observed at 0.1 μM (31.8 ± 4.17 pg/mL, *p* < 0.05), 0.3 μM (24.9 ± 2.88 pg/mL, *p* < 0.01), and 1 μM (19.8 ± 0.32 pg/mL, *p* < 0.01). These findings demonstrate that both CMX and brevilin A significantly inhibit TNF-α-induced PGE_2_ production, further supporting their anti-inflammatory potential.

Taken together, these results suggest that CMX and brevilin A effectively suppressed the overproduction of NO and PGE_2_, two key inflammatory mediators in TNF-α-stimulated NHDFs. These inhibitory effects highlight the anti-inflammatory properties of CMX, with brevilin A identified as the active constituent responsible for mitigating inflammation-induced skin damage.

### 3.3. Effects of Brevilin A on TNF-α-Stimulated Phosphorylation of Mitogen-Activated Protein Kinases (MAPKs) in NHDFs

To investigate the molecular mechanisms underlying the protective effects of brevilin A, the phosphorylation levels of mitogen-activated protein kinases (MAPKs), including extracellular signal-regulated kinase (ERK), c-Jun N-terminal kinase (JNK), and p38, were analyzed using Western blotting.

**Figure 6:**
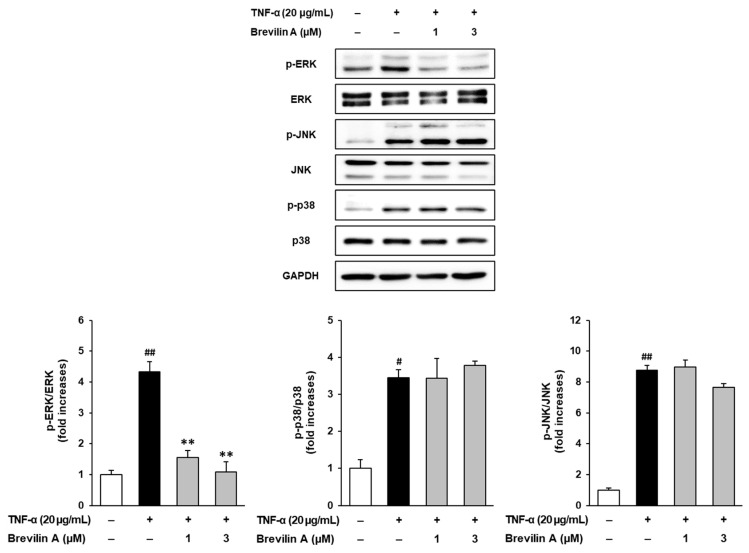
Effects of brevilin A on MAPK phosphorylation. NHDF cells were dispensed into a 6-well plate at a concentration of 3 × 10^5^. The cell cycle was synchronized by incubating the cells in an FBS-free medium for 24 h. Next, the cells were pretreated with various concentrations of brevilin A, followed by the addition of TNF-α. After 15 min, samples were collected. The collected samples were quantified for protein content and subjected to Western blot analysis to detect the protein bands of ERK, JNK, and p38. The experimental results are presented as S.E.M., and statistical significance is indicated as follows: *p* < 0.05 (#) and *p* < 0.01 (##) compared to the control group and *p* < 0.01 (**) compared to the TNF-α-induced group.

As shown in Figure 6, TNF-α stimulation significantly increased the phosphorylation of ERK, JNK, and p38, indicating the activation of the MAPK signaling pathway. Among these, ERK phosphorylation was particularly upregulated, with a 4.32 ± 0.34-fold (*p* < 0.01) increase compared to the untreated control group. Brevilin A treatment selectively inhibited ERK phosphorylation in a dose-dependent manner. At 1 μM, brevilin A reduced ERK phosphorylation to 1.55 ± 0.23-fold (*p* < 0.05), while, at 3 μM, the phosphorylation levels were further decreased to 1.09 ± 0.32-fold (*p* < 0.05). In contrast, brevilin A treatment did not significantly alter JNK and p38 phosphorylation.

These findings suggest that brevilin A exerts protective effects against TNF-α-induced ECM degradation and inflammatory responses by selectively inhibiting ERK phosphorylation. By modulating the MAPK signaling pathway, brevilin A may play a crucial role in preventing collagen degradation and inflammatory damage in NHDFs.

## 4. Discussion

The aging of the skin is a common concern in dermatology. With increasing interest in personal well-being, consumers demand products containing natural bioactive ingredients to support skin health [23]. Natural compounds such as flavonoids, sesquiterpene lactones, and polyphenols have demonstrated anti-skin aging effects [24,25,26,27,28,29]. Among them, sesquiterpene lactones have shown activity in NHDFs [30,31]; thus, understanding their function is important in protecting the skin from aging. In this study, we evaluated the protective effects of sesquiterpene lactone brevilin A and its source plant, *Centipeda minima* (CMX), on TNF-α-stimulated NHDFs (Figure 1).

UV-induced intracellular ROS and pro-inflammatory cytokines such as TNF-α are key mediators of skin aging. Mitochondrial ROS further amplify TNF-α production, contributing to ECM degradation and inflammation-related skin damage [1,6,8,9]. As shown in Figure 4A, CMX and brevilin A significantly inhibited ROS accumulation in TNF-α-stimulated NHDFs, supporting their antioxidant roles.

TNF-α is secreted by skin fibroblasts and keratinocytes and plays a critical role in photoaging by inducing enzymes such as MMP-1, which degrade collagen in the dermal ECM [18,19]. The resulting collagen loss contributes to the wrinkling and sagging of the skin. Our results showed that CMX and brevilin A suppressed MMP-1 expression and restored COLIA1 secretion in TNF-α-treated NHDFs (Figure 4B,C), suggesting their anti-wrinkle potential and identifying brevilin A as an active component.

In addition to ECM damage, TNF-α induces inflammatory mediators such as nitric oxide (NO) and prostaglandin E2 (PGE2), which contribute to chronic skin inflammation and aging [12,32,33]. Treatment with CMX and brevilin A significantly reduced the TNF-α-induced overproduction of NO and PGE2 in NHDFs (Figure 5), reinforcing their anti-inflammatory efficacy.

MAPK pathway activation is involved in TNF-α-induced MMP-1 expression and inflammation [34,35]. Brevilin A selectively inhibited ERK phosphorylation (Figure 6) without affecting JNK or p38, consistent with previous studies suggesting its specificity for the ERK pathway [36,37]. This suggests that brevilin A may exert anti-aging effects by targeting ERK signaling directly or through upstream regulators, such as MEK1/2 or redox-sensitive mediators.

This study had several limitations. First, only one skin cell type (NHDFs) was used, which may not fully represent the complex skin microenvironment. Second, UVB-induced models that better simulate environmental aging were not included. Third, our assays were limited to short-term in vitro settings, lacking gene expression profiling or long-term evaluation. These limitations should be addressed in future studies.

Moreover, previous studies have shown that TNF-α-induced skin inflammation activates transcription factors such as NF-κB and AP-1 [13,34]. Although our study focused on the ERK arm of the MAPK pathway, future investigations should examine the effects of CMX and brevilin A on these additional signaling pathways to provide a more comprehensive understanding of their anti-inflammatory mechanisms [38].

In summary, our findings demonstrate that *Centipeda minima* (CMX) and brevilin A mitigated TNF-α-induced oxidative stress and inflammatory damage in NHDFs by reducing the intracellular ROS, NO, and PGE_2_ levels; downregulating MMP-1; and restoring collagen synthesis. Mechanistically, brevilin A inhibits ERK activation, thereby preserving ECM integrity. These results suggest that CMX and brevilin A are promising agents for natural anti-aging skin therapy.

To overcome the limitations of the current in vitro model, we plan to perform in vivo studies using UVB-induced skin aging mouse models to assess the antioxidant and anti-inflammatory efficacy of CMX and brevilin A, as well as their histological and pharmacokinetic profiles, under physiologically relevant conditions. In addition, advanced methodologies such as three-dimensional organotypic skin models and co-culture systems incorporating keratinocytes and fibroblasts will be employed to better replicate the complex skin microenvironment and elucidate the intercellular signaling mechanisms involved in ECM preservation and inflammation control.

## 5. Conclusions

**Figure 7:**
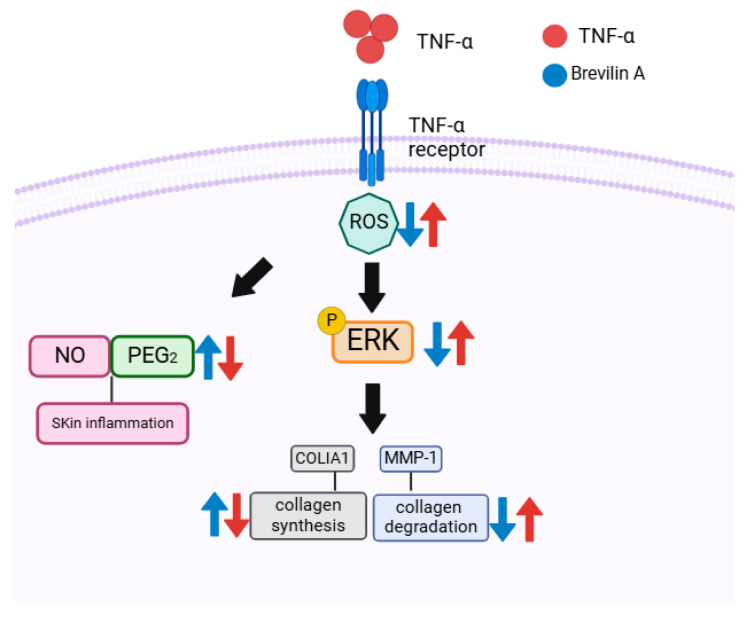
Illustration of the potential protective role of brevilin A in TNF-α-induced NHDFs.

This study demonstrated that *Centipeda minima* (CMX) and its active compound brevilin A exerted protective effects against TNF-α-induced damage in normal human dermal fibroblasts (NHDFs). CMX and brevilin A significantly inhibited TNF-α-induced increases in the intracellular ROS, NO, and PGE2 levels, thereby mitigating oxidative stress and inflammation. Additionally, they reduced MMP-1 expression while restoring the COLIA1 levels, thereby preserving the extracellular matrix (ECM) and counteracting the skin aging processes. Mechanistically, brevilin A inhibited ERK phosphorylation within the MAPK signaling pathway, suggesting its role in preventing TNF-α-induced ECM degradation and inflammation (Figure 7).

These findings indicate that CMX and brevilin A are potential natural anti-aging agents that protect the skin from oxidative stress and inflammatory responses. Although this study focused on cellular efficacy, future work will explore formulation challenges associated with incorporating CMX and brevilin A into topical products. In particular, we plan to evaluate the compound stability, dermal permeability, and delivery efficiency using three-dimensional (3D) skin models to support their pharmaceutical and cosmetic applications. Furthermore, future studies will include in vivo validation using animal models and the use of other skin cell types to fully elucidate their protective effects and therapeutic potential. Overall, CMX and brevilin A are promising candidates for the alleviation of skin aging and inflammation.

## Data Availability

The datasets produced in this study can be obtained from the corresponding author upon reasonable request.

## Abbreviations

COLIA1	Collagen Type I Alpha 1
COX-2	Cyclooxygenase 2
DCFDA	2′,7′-Dichlorodihydrofluorescein Diacetate
ERK	Extracellular Signal-Regulated Kinase
iNOS	Inducible Nitric Oxide Synthase
JNK	Jun N-terminal Kinase
MAPKs	Mitogen-Activated Protein Kinases
MMP-1	Matrix Metallopeptidase 1
NHDFs	Normal Human Dermal Fibroblasts
NO	Nitric Oxide
P38	p38 Mitogen-Activated Protein Kinase
PGE2	Prostaglandin-E2
ROS	Reactive Oxygen Species
TNF-α	Tumor Necrosis Factor-Alpha
UV	Ultraviolet
